# Forecast and verification of the active compounds and latent targets of Guyuan decoction in treating frequently relapsing nephrotic syndrome based on network pharmacology

**DOI:** 10.1080/0886022X.2023.2184654

**Published:** 2023-03-03

**Authors:** Haiyun Wang, Junjie Shi, Binbin Tang, Yanfeng Liu, Qili Wang

**Affiliations:** a Traditional Chinese Medicine Pediatrics Department, Second Outpatient Department, Tongde Hospital of Zhejiang Province, Hangzhou, China; b Clinical School of Medicine, Hangzhou Medical College, Hangzhou, China; c Internal Medicine of Traditional Chinese Medicine Department, Second Outpatient Department, Tongde Hospital of Zhejiang Province, Hangzhou, China; d Traditional Chinese Medicine Department, Jinan Maternal and Child Health Hospital Shunyu Road Community Health Service Center, Jinan, China; e Pediatrics Department, The First Affiliated Hospital of Zhejiang Chinese Medical University (Zhejiang Provincial Hospital of Traditional Chinese Medicine), Hangzhou, China

**Keywords:** Guyuan decoction, frequently relapsing nephrotic syndrome, network pharmacology, active compounds, significant pathways

## Abstract

**Background:**

Our study majorly utilizes network pharmacology combined with molecular docking to explore the latent active components and associated pivotal targets of Guyuan Decoction (GYD) in the treatment of frequently relapsing nephrotic syndrome (FRNS).

**Methods:**

All active components and latent targets of GYD were retrieved from TCMSP database. The target genes for FRNS in our research were obtained from the GeneCards database. The drug-compounds-disease-targets (D-C-D-T) network was established using Cytoscape 3.7.1. STRING database was applied to observe the protein interaction. Pathway enrichment analyses (GO and KEGG) were conducted in R software. Moreover, molecular docking was employed to further validate the binding activity. MPC-5 cells were treated with adriamycin to mimic FRNS *in vitro* and to determine the effects of luteolin on modeled cells.

**Results:**

A total of 181 active components and 186 target genes of GYD were identified. Meanwhile, 518 targets related to FRNS were also revealed. Based on the intersection using a Venn diagram, 51 common latent targets were recognized to be associated with active ingredients and FRNS. Additionally, we identified the biological processes and signaling pathways involved in the action of these targets. Molecular docking analyses illustrated that AKT1 and CASP3 interacted with luteolin, wogonin, and kaempferol, respectively. Moreover, luteolin treatment enhanced the viability but inhibited the apoptosis of adriamycin-treated MPC-5 cells *via* regulating AKT1 and CASP3.

**Conclusion:**

Our study forecasts the active compounds, latent targets, and molecular mechanisms of GYD in FRNS, which helps us to understand the action mechanism of GYD in FRNS comprehensive treatment.

## Introduction

Nephrotic syndrome is the most common glomerular disease in childhood, with an average incidence of 2–16.9 per 100,000 children worldwide [1]. The major clinical manifestations of this disease are massive proteinuria, hypoalbuminemia, hyperlipidemia, and various degrees of edema [2]. The altered glomerular filtration membrane permeability and massive urinary protein production resulting from various causes are the main characteristics and triggers, and a great number of urinary proteins can give rise to hypertension and hyperlipidemia, which contribute to glomerulosclerosis [3]. The pathogenesis of nephrotic syndrome has not been fully elucidated, which is currently believed to be mainly implicated in immune imbalance, systemic circulating factors, and podocyte gene mutations. Since the 1950s, glucocorticoids have been considered as the first-line treatment for children with nephrotic syndrome [4]. According to the guidelines issued by the organization for improving the prognosis of patients with kidney disease worldwide, frequent relapsing nephrotic syndrome (FRNS) is defined as those with more than 2 relapses within 6 months of hormone therapy or more than 4 relapses within 1 year [5]. Children with FRNS are often required to be treated with other immunosuppressants, such as cyclophosphamide, calcineurin inhibitors, mycophenolate mofetil, cyclosporine A, and rituximab [6]. However, in addition to the adverse reactions of these drugs, some children are still unable to achieve complete remission or facing the challenge of frequent recurrence, and eventually progress to end-stage renal disease [7,8]. As such, finding vicarious treatments with low toxicity is of vital clinical significance to facilitate the curative therapy of FRNS and ameliorate side effects.

As one of the most popular complementary and alternative medicine models in China, traditional Chinese medicine (TCM) is gradually accepted by foreigners due to its obvious curative effect, abundant resources, and low toxicity, which is increasingly used in Western countries [9]. Guyuan decoction (GYD) is a classic prescription for the clinical treatment of various diseases in China, like FRNS and other kidney diseases [10–12]. GYD is composed of *Astragali Radix* (Huangqi), *Pseudostellariae Radix* (Taizishen), *Rhizoma Atractylodis Macrocephalae* (Baizhu), *Poria cocos* (Fuling), *Saposhnikoviae Radix* (Fangfeng), *Glycyrrhizae Radix et Rhizoma* (Gancao), *Phellodendri Chinensis Cortex* (Huangbai), *Amomi Fructus* (Sharen), and *Stigma maydis* (Yumixu). Previously, Wen X et al. analyzed the latent targets and molecular mechanisms of *Astragali Radix* in the treatment of nephrotic syndrome using network pharmacology [13]. Another study also reported that *Poria cocos* improved nephrotic syndrome in rats *via* restraining water and sodium channels [14]. Based on the description of Xia P et al. *Astragali Radix*, *Pseudostellariae Radix*, *Rhizoma Atractylodis Macrocephalae*, and *Poria cocos* are the most commonly used core herbal medicine for the treatment of chronic kidney disease [15]. These aforementioned studies thus illuminated that various TCMs in GYD are conducive to the treatment of nephrotic syndrome. However, the main active components of GYD and the relevant mechanisms of pharmacological anti-FRNS effects remain vague, thus warranting further elucidation.

Network pharmacology, which is firstly proposed by Andrew L Hopkins, a pharmacologist at Dundee University in 2007, is a new discipline based on systems biology, computer technology, network biology, and other emerging technologies to select specific signal nodes for multi-target drug molecular design [16]. Network pharmacology provides the possibility to comprehensively explore the mechanism of TCM in treating diseases, which coincides with the concept of syndrome differentiation, system, and overall research of traditional medicine [17]. As a helpful tool, network pharmacology offers a new chance to gain further insight on the interaction between the active ingredients and associated targets, with a highlight on the mechanisms of action as well [18].

This study, accordingly, intends to screen the main active components of GYD and predicts its targets based on the method of network pharmacology, while constructing the visualized network relationship of drug-components-disease-targets (D-C-D-T) at the same time. By doing so, some theoretical support for more comprehensive and in-depth exploration of the pharmacodynamic material basis and molecular mechanism of GYD can be provided, which, we hope, can contribute to the clinical application and further experimental research of GYD.

## Material and methods

### Collection of chemical ingredients

Nine TCM compounds in GYD were obtained from the TCMSP database (http://tcmspw.com/tcmsp.php). In combination with the analyses of the existing literature, 6 of 9 compounds were selected finally, namely, atractylenolide, atractylon, prim-o-glucosylcimifugin, astragaloside, glycyrrhizic acid, and glycyrrhetinic acid [19–22].

### Screening of active compounds

The TCMSP database was applied to screen the active compounds based on the absorption, distribution, metabolism, and excretion (ADME) criteria, followed by the prediction on the targets of all compounds [23]. For the follow-up studies, the selected candidate ingredients in TCMSP must meet the requirements of oral bioavailability (OB) ≥ 30% and drug-likeness (DL) ≥ 0.18.

### Prediction of target genes associated with the active components of GYD

The genes related to the active components of GYD in this study were retrieved from the TCMSP database under the condition of ‘*Homo sapiens*’. We also used the UniProt database (http://www.uniprot.org/) to correct and unify the names of these targets, and then applied the Swiss Target Prediction database (http://www.swisstargetprediction.ch/) to supplement the information of these target genes.

### Collection of disease-targets

The target genes for FRNS in our research were also obtained from the GeneCards database (https://www.genecards.org/) using the keyword of ‘frequently relapsing nephrotic syndrome’.

### Intersection of chemical ingredient-targets and disease-targets

On the Venny2.1 online software mapping tool platform (https://bioinfogp.cnb.csic.es/tools/venny/), we inputted the targets of chemical components and FRNS, respectively. A Venn diagram was subsequently drawn to obtain the common targets of components and FRNS.

### Construction of network diagram

The tabulation of ‘compounds to disease’ has been introduced into Cytoscape 3.7.1 software. Next, the names of GYD compounds and FRNS were imported into Cytoscape to establish the network diagram of D-C-D-T. The interaction of D-C-D-T was shown in network diagram through nodes and edges, where nodes represented molecules or target proteins and edges denoted the interaction among compounds, disease, and targets.

### Construction of protein-protein interaction (PPI) network

The association in the retrieved targets of GYD active ingredients and FRNS was analyzed through STRING database (https://string-db.org/). In detail, for the construction of a PPI network, the species restriction was set to ‘*Homo sapiens*’, and the required minimum interaction score > 0.4 was selected. The R package was exploited to screen central proteins and rank them according to the degree of association between proteins.

### Pathway enrichment analysis

We installed the Bioconductor package ‘org.Hs.eg.db’ in R software. The common target(s) of ingredients and FRNS was/were transformed into entrezID. Then, we installed ‘clusterProfiler’ package in R software. Gene Ontology (GO) and Kyoto Encyclopedia of Genes and Genomes (KEGG) functional enrichment analyses on the key target genes were performed at *p* < 0.05 and *Q* < 0.05 according to the transformed entrezID.

### Molecular docking

In the target interaction network obtained from PPI analysis, 2 targets with maximum count scores were selected, namely, AKT1 and CASP3. Thereafter, we verified the compound-target correlation with the help of molecular operating environment (MOE) software (v2015.10). Then, PDB database (http://www.rcsb.org/pdb/home/home.do) was taken to acquire the 3D structure of proteins.

### Cell culture and treatment

Mouse kidney podocytes MPC-5 (CTCC-400-0327) purchased from Meisen Chinese Tissue Culture Collections (China) was maintained in RPMI-1640 medium (PM150110, Procell, China) containing 10% fetal bovine serum (164210-50, Procell, China) and 1% penicillin-streptomycin solution (PB180120, Procell, China) at 37 °C with 5% CO_2_.

To mimic FRNS *in vitro*, MPC-5 cells were stimulated with 10 µM adriamycin (ADR, T1020, TopScience, China) for 6 h (h).

### Cell counting kit-8 (CCK-8) assay

MPC-5 cells were seeded into a 96-well plate at a density of 1 × 10^4^ cells/well and treated with 100, 150, 200 or 250 μM luteolin (LUT, HY-N0162, MedChemExpress, China) for 48 h to screen out the appropriate drug concentration. After the completion of drug treatment, 10 μL of CCK-8 reagent (GK10001, GLPBIO, USA) was added to incubate with the MPC-5 cells. After the reaction time of 4 h, the 96-well plate was placed under a microplate reader (ReadMax 1200, Shanpu Biotech, China) to test the absorbance (at 450 nm) of cells under different treatment conditions.

Thereafter, CCK-8 assay was performed again to determine the effect of LUT (100, 150, or 200 μM) on the viability of ADR-treated MPC-5 cells.

### Flow cytometry

Annexin V-FITC Apoptosis Detection Kit (C1062) available from Beyotime (China) was utilized to test the apoptosis of cells. MPC-5 cells were resuspended in 195 μL Annexin V-FITC binding buffer, reacted with 5 μL Annexin V-FITC and mixed with 10 μL propidium iodide (PI) staining solution for 20 min (min). Finally, the cell apoptosis was detected using the flow cytometer (CytoFLEX, Beckman Coulter, USA).

### Protein extraction and Western blot detection

Total protein in MPC-5 cells was extracted using the RIPA reagent (89901, Thermo Scientific, USA). Then, the concentration of protein samples was quantified by BCA protein quantitation kit (55 R-1544, Fitzgerald, USA). Next, sodium dodecyl sulfate-polyacrylamide gel electrophoresis (SDS-PAGE) gels were exploited to separate the protein sample, which was then moved to polyvinylidene fluoride membranes (24937, Sigma-Aldrich, China). The membranes were sequentially soaked in blocking solution to reduce nonspecific binding and incubated with primary antibodies against Cleaved (Clea)-caspase-3 (#9661, 1:1000, 17, 19 kDa, Cell Signaling Technology, USA), AKT1 (ab179463, 1:10,000, 56 kDa, abcam, UK), phosphorylated (p)-AKT1 (ab81283, 1:10,000, 56 kDa, abcam, UK), and GAPDH (ab9485, 1:2500, 37 kDa, abcam, UK) at 4 °C overnight. Next, the membranes were incubated with the secondary antibodies including goat anti-rabbit IgG (ab97051, 1:5000, abcam) and rabbit anti-mouse IgG (ab6709, 1:2000, abcam) for 2 h. Excellent Chemiluminescent Substrate Detection Kit (E-BC-R347, Elabscience, China) was used to measure the protein bands, and ImageQuant LAS 4000MINI Ultra-Sensitive Chemiluminescence Imager (Sinopharm Chemical Reagent Co. Ltd., China) was employed to scan the bands. GAPDH served as the loading control of this assay.

### Statistical analysis

All results from at least triplicate experiments were expressed as mean ± standard deviation. All statistical analyses were carried out using SPSS 20.0 (IBM, USA). One-way ANOVA was used to evaluate the significance among multiple groups, followed by the Bonferroni *post hoc* test. The data with *p* < 0.05 were deemed to be statistically significant.

## Results

### Screening of active ingredients and targets

The thresholds of OB ≥ 30% and DL ≥ 0.18 were set in the TCMSP database to screen the active ingredients of 9 TCMs in GYD. In this study, atractylenolide, atractylon, prim-o-glucosylcimifugin, astragaloside, glycyrrhizic acid, and glycyrrhetinic acid were selected, and a total of 181 active ingredients were obtained. Next, we inputted the targets corresponding to the active ingredients into the UniProt database and used the Swiss Target Prediction database to supplement, delete, and deduplicate the target information. A sum of 186 drug targets was finally screened out. The number of components and predicted targets corresponding to various TCMs is presented in Table 1.

**Table 1.** Statistics of basic information of traditional Chinese medicine-ingredient-target.

TCM name	Number of ingredients	Number of predicted targets
Baizhu	5	22
Fangfeng	16	233
Fuling	13	26
Taizishen	6	122
Huangqi	20	298
Huangbai	35	357
Sharen	8	59
Yumixu	8	94
Gancao	89	1541
Buchong	3	30

### Potential targets of FRNS

In this research, we searched the database of GeneCards and obtained 518 potential targets following the removal of duplicate targets. These targets were strongly associated with the initiation and development of FRNS.

### Screening of the overlapping gene

**Figure 1.**
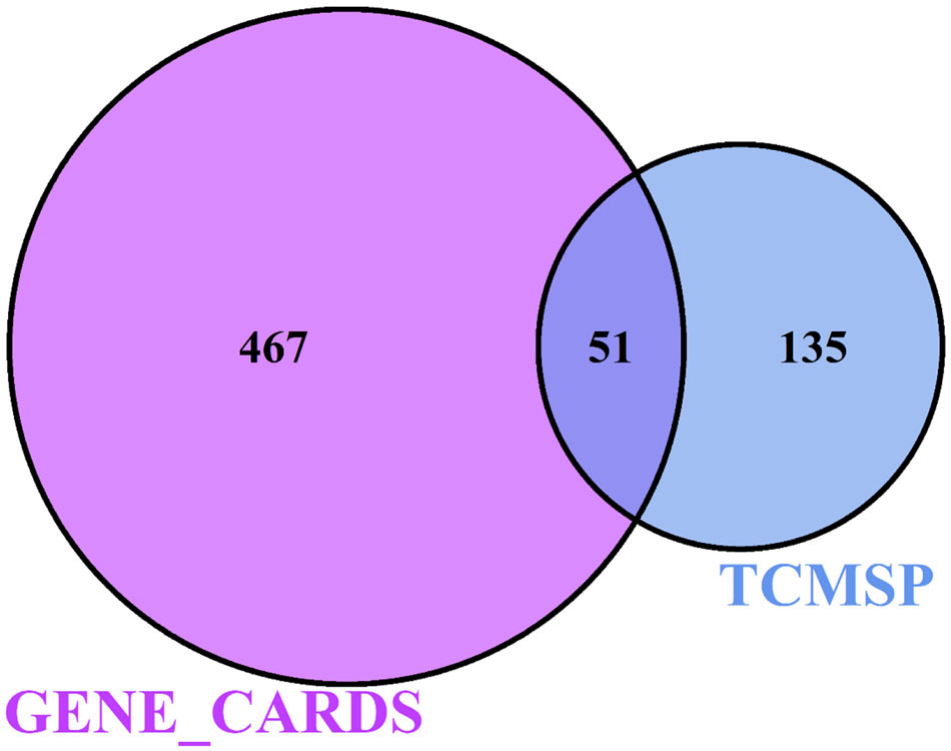
Venn diagram of 51 latent common targets.

A Venn diagram was drawn by inputting 186 drug targets and 518 FRNS targets into the online software Venny2.1. The generated Venn diagram suggested that 51 common potential targets were related to the active ingredients and FRNS (Figure 1).

### Construction of D-C-D-T network

**Figure 2.**
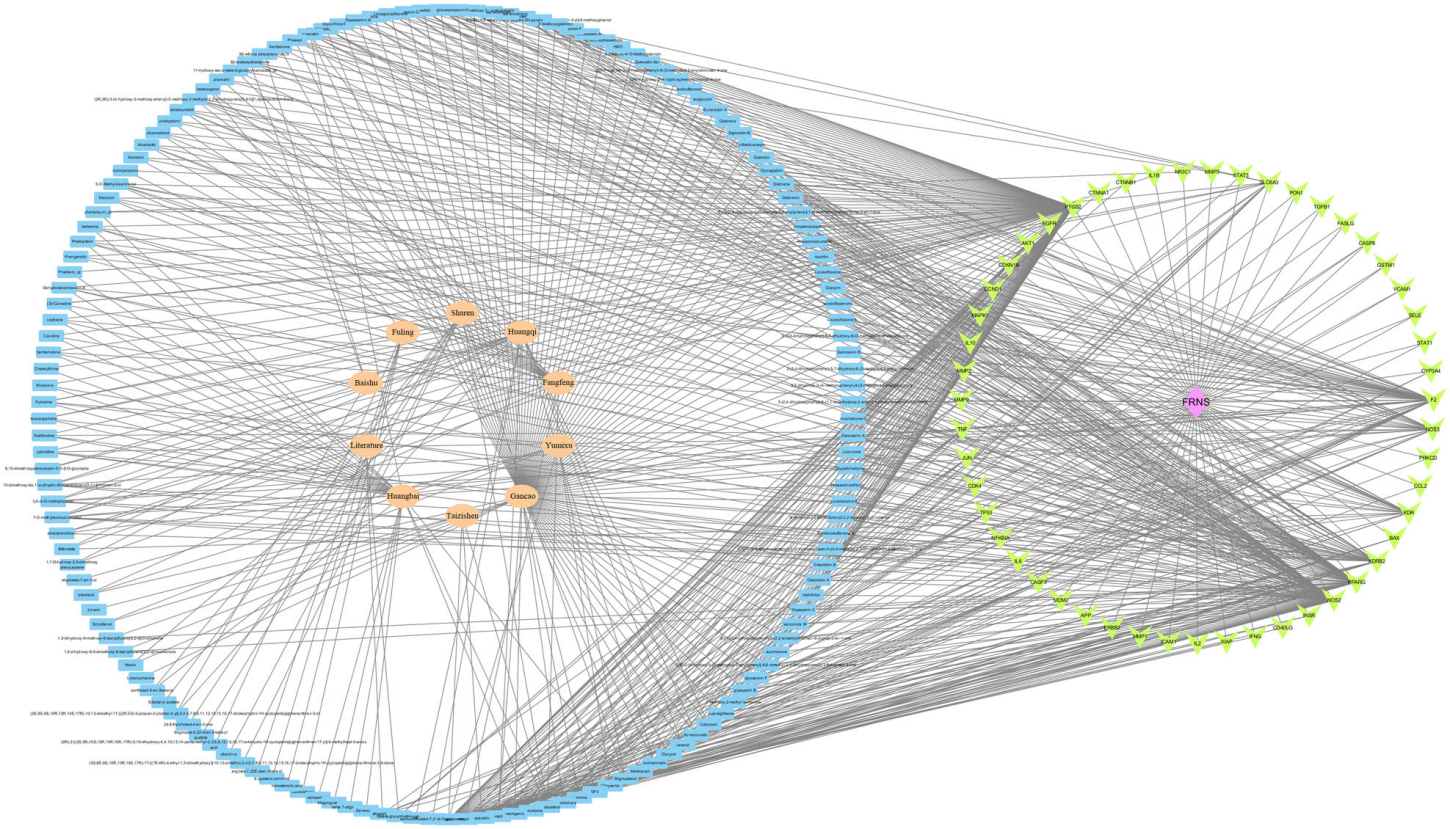
A drug-components-disease-targets network of four parts. After inputting the main active components of each herb in Guyuan decoction (GYD) and the 51 latent common targets obtained, Cytoscape 3.7.1 software was used to construct the ‘drug-components-disease-targets’ interaction network diagram. FRNS: frequently relapsing nephrotic syndrome; green: 51 latent common targets; pink: FRNS targets; flesh color: Chinese herbal medicine; blue: active ingredients of GYD.

To explore the latent mechanism underlying the functions of GYD on FRNS, we established a D-C-D-T network utilizing Cytoscape 3.7.1 software (Figure 2).

### Analysis of compound-FRNS PPI network

**Figure 3.**
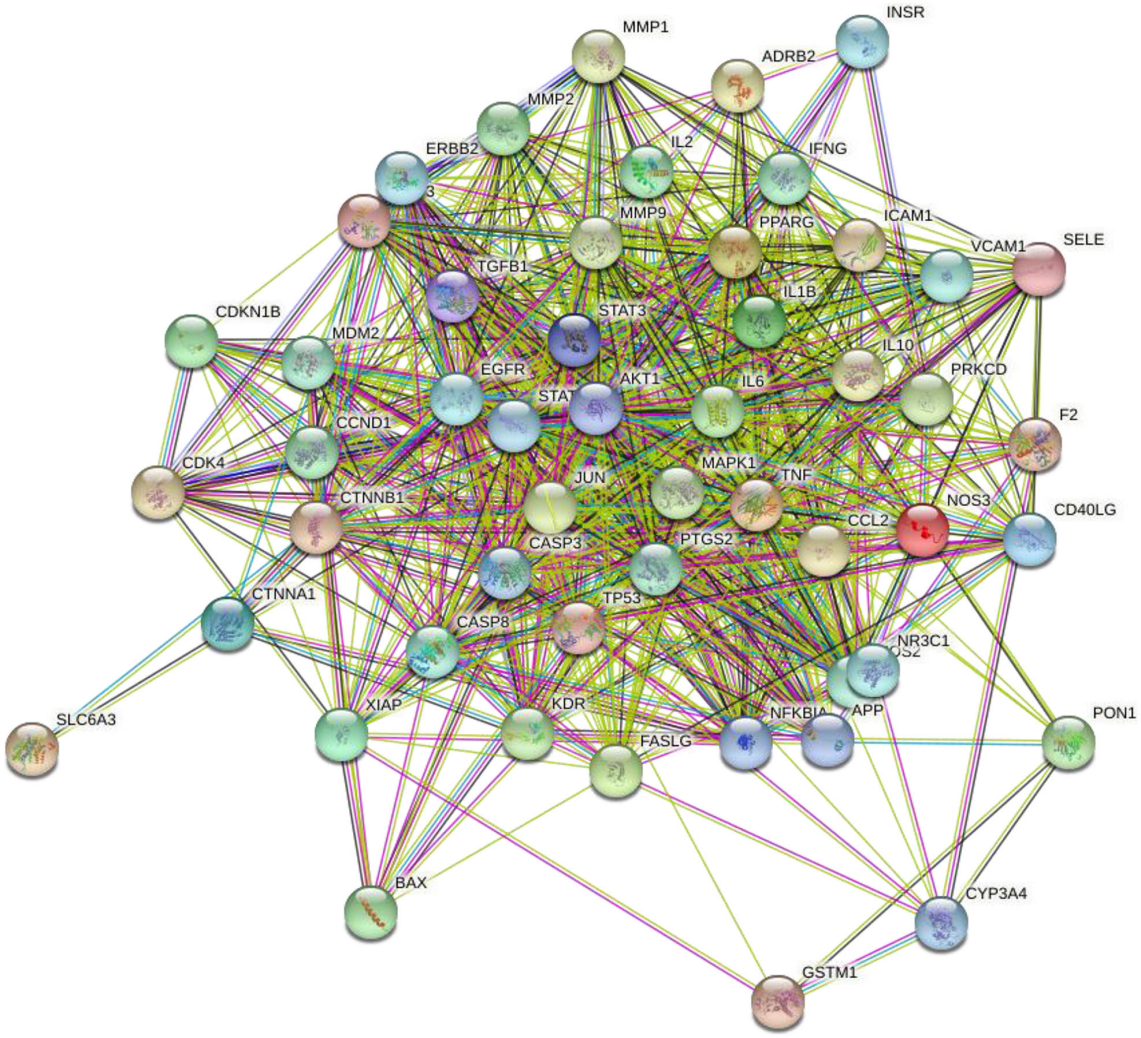
Protein-protein interaction (PPI) network of 51 overlapping genes.

**Figure 4.**
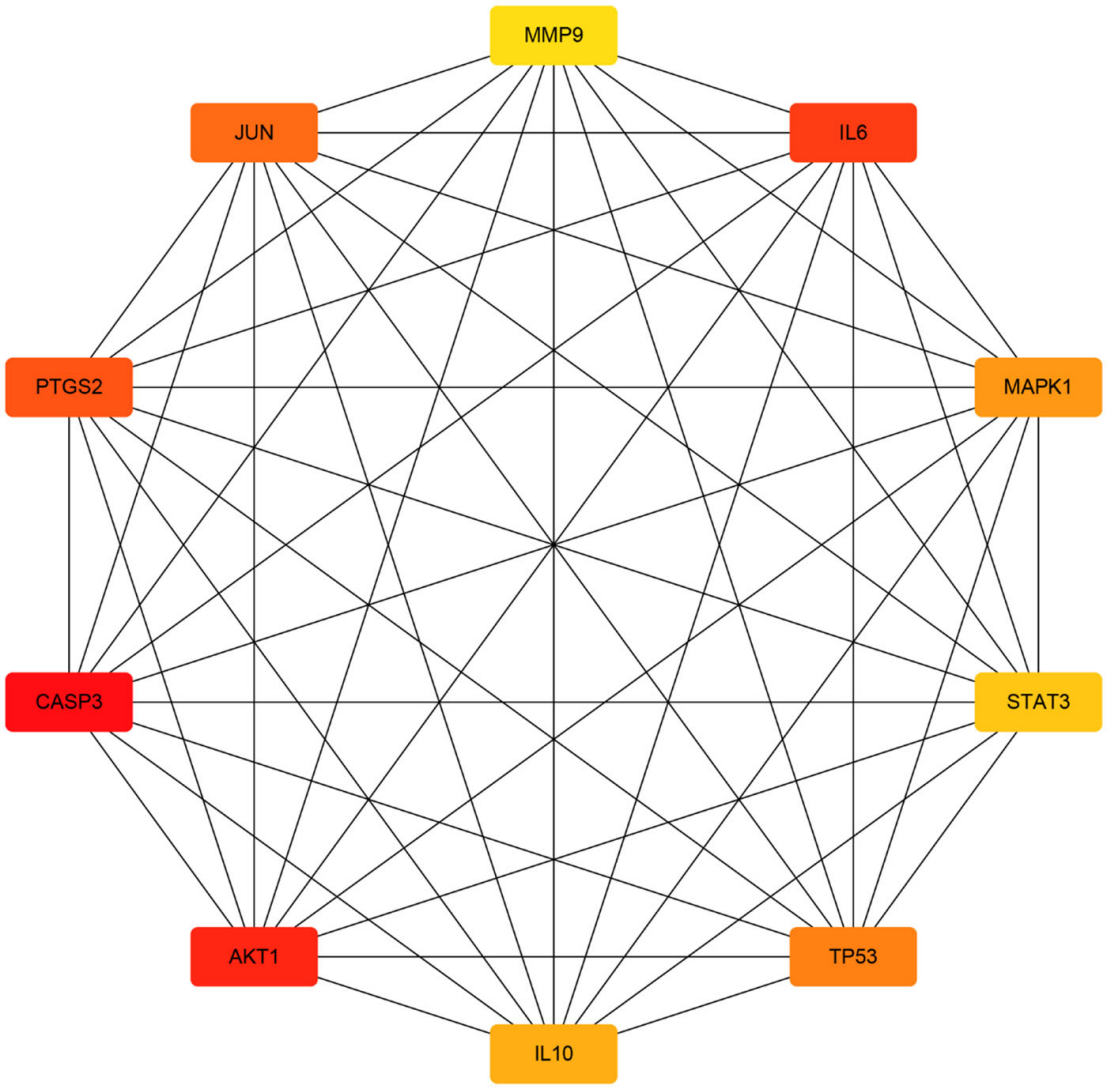
Top 10 targets from PPI network.

To elucidate the mechanism accounting for the interaction of these overlapping genes, the aforementioned 51 common targets were analyzed in the STRING database, and then a PPI network was correspondingly constructed (Figure 3). Each edge represented the interaction between proteins. More lines indicated the greater degree of association. In addition, the top 10 genes tightly related to other genes are shown in Figure 4. Red indicated the highest degree and yellow meant the lowest degree. These genes were AKT1, CASP3, IL6, PTGS2, JUN, MAPK1, TP53, IL10, STAT3, and MMP9.

### Molecular function and pathway prediction

**Figure 5.**
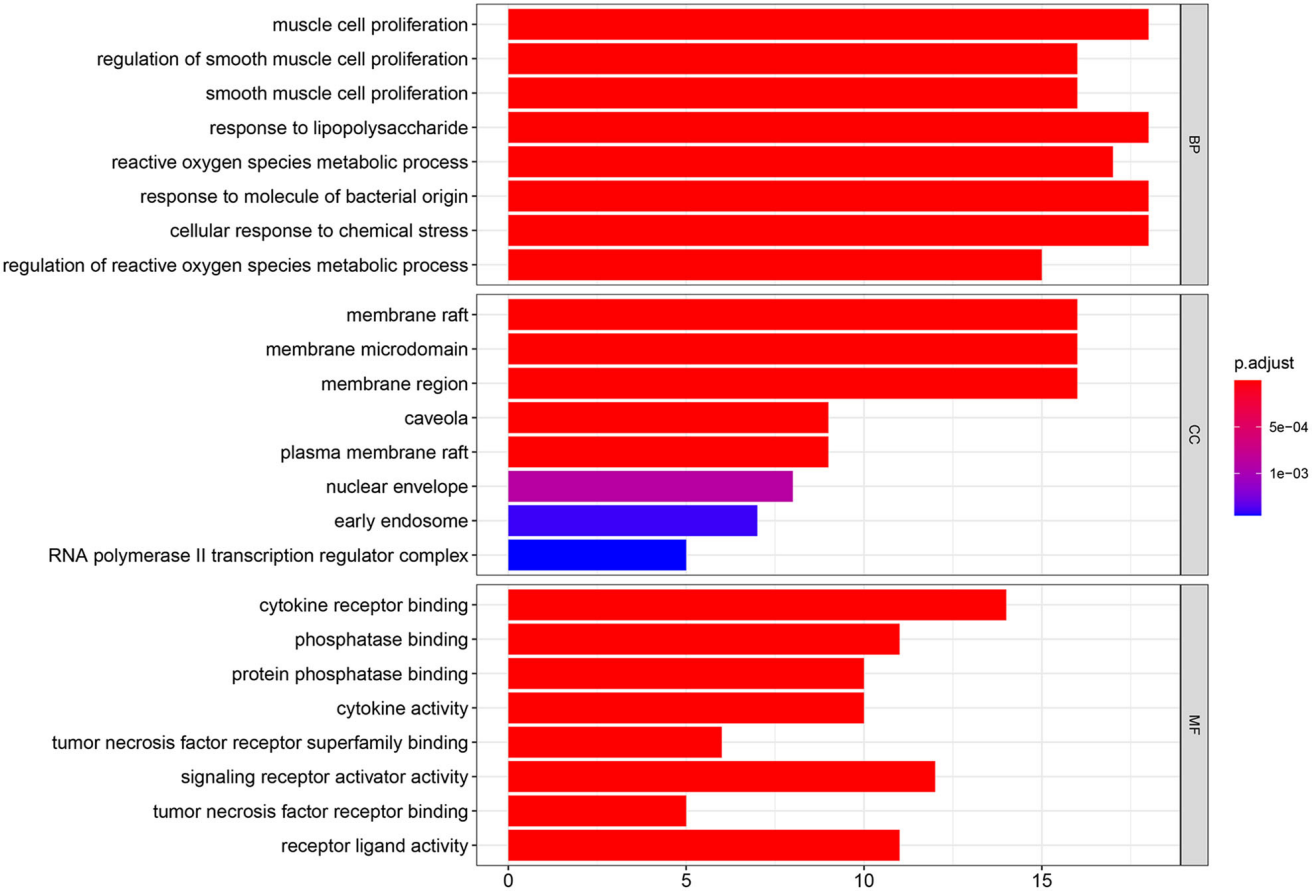
Gene Ontology enrichment analysis of 51 overlapping genes.

**Figure 6.**
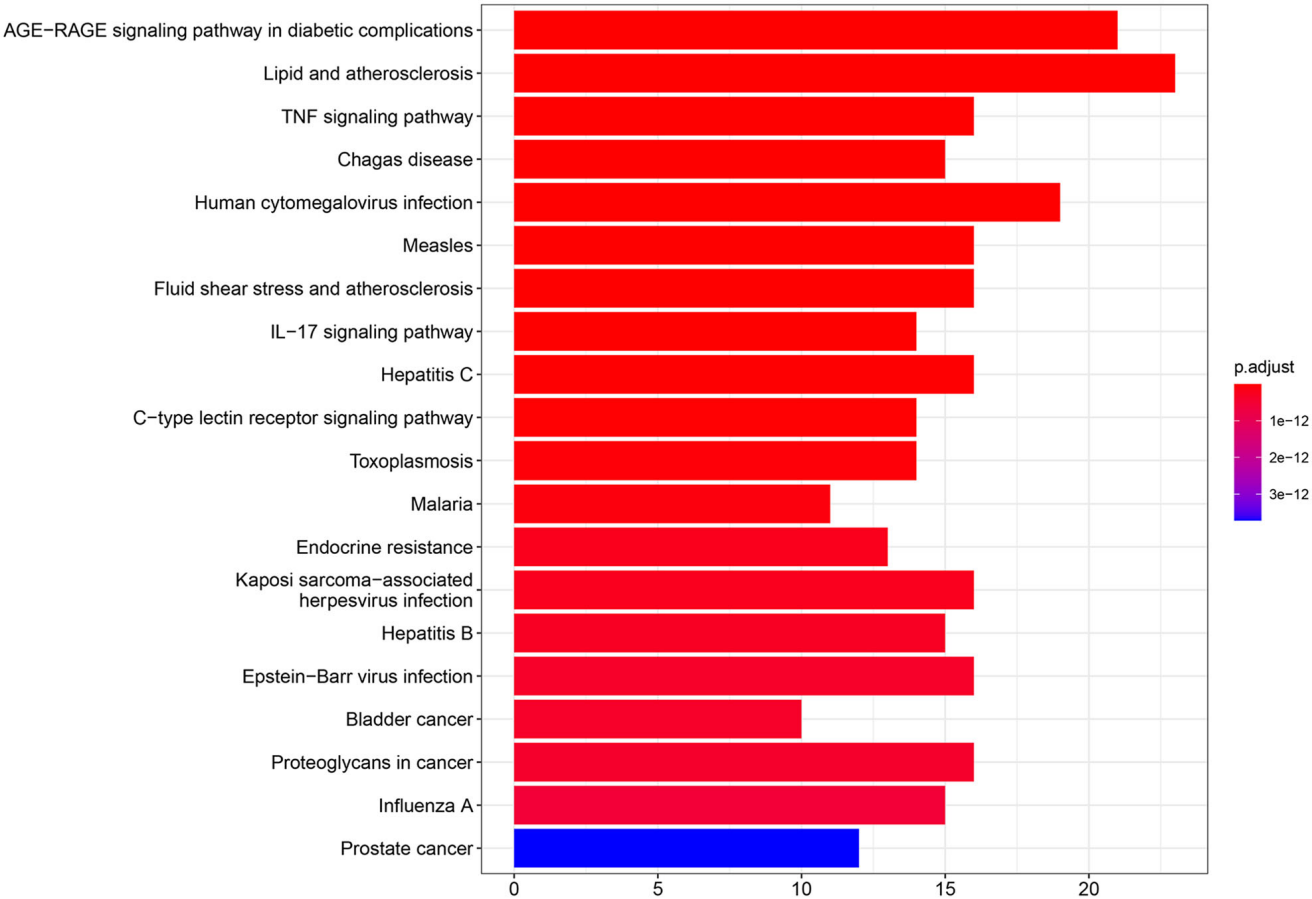
Top 20 pathways from Kyoto Encyclopedia of Genes and Genomes (KEGG).

In order to clarify the effect and underlying mechanism of these GYD active components on FRNS, we selected three parts of biological process (BP), cellular composition (CC), and molecular function (MF) from 51 common targets by GO analysis after the processing with R language. The top 8 enriched GO terms were confirmed by GO analysis, as presented in the bar plot (Figure 5). BP enrichment was correlated with muscle cell proliferation, regulation of smooth muscle cell proliferation, smooth muscle cell proliferation, response to lipopolysaccharide, reactive oxygen species metabolic process, etc. (Figure 5). Besides, most GO terms of CC were mainly involved in membrane raft, membrane microdomain, membrane region, caveola, plasma membrane raft, etc. (Figure 5). In addition, MF enrichment was related to cytokine receptor binding, phosphatase binding, protein phosphatase binding, cytokine activity, tumor necrosis factor receptor superfamily binding, etc. (Figure 5). To further illustrate how GYD affects FRNS by these latent targets, the top 20 pathways were singled out based on the *p*-value from small to large (Figure 6). The first three entries of enrichment were the AGE-RAGE signaling pathway in diabetic complications, lipid and atherosclerosis, and TNF signaling pathway. *p* represented the significance of enrichment, and the redder the color, the higher the significance.

### Compound-major target molecular docking

Subsequently, we selected the first two targets (AKT1 and CASP3) with high scores from 51 latent targets for molecular docking with 3 active components of GYD (luteolin, wogonin, and kaempferol).

**Figure 7.**
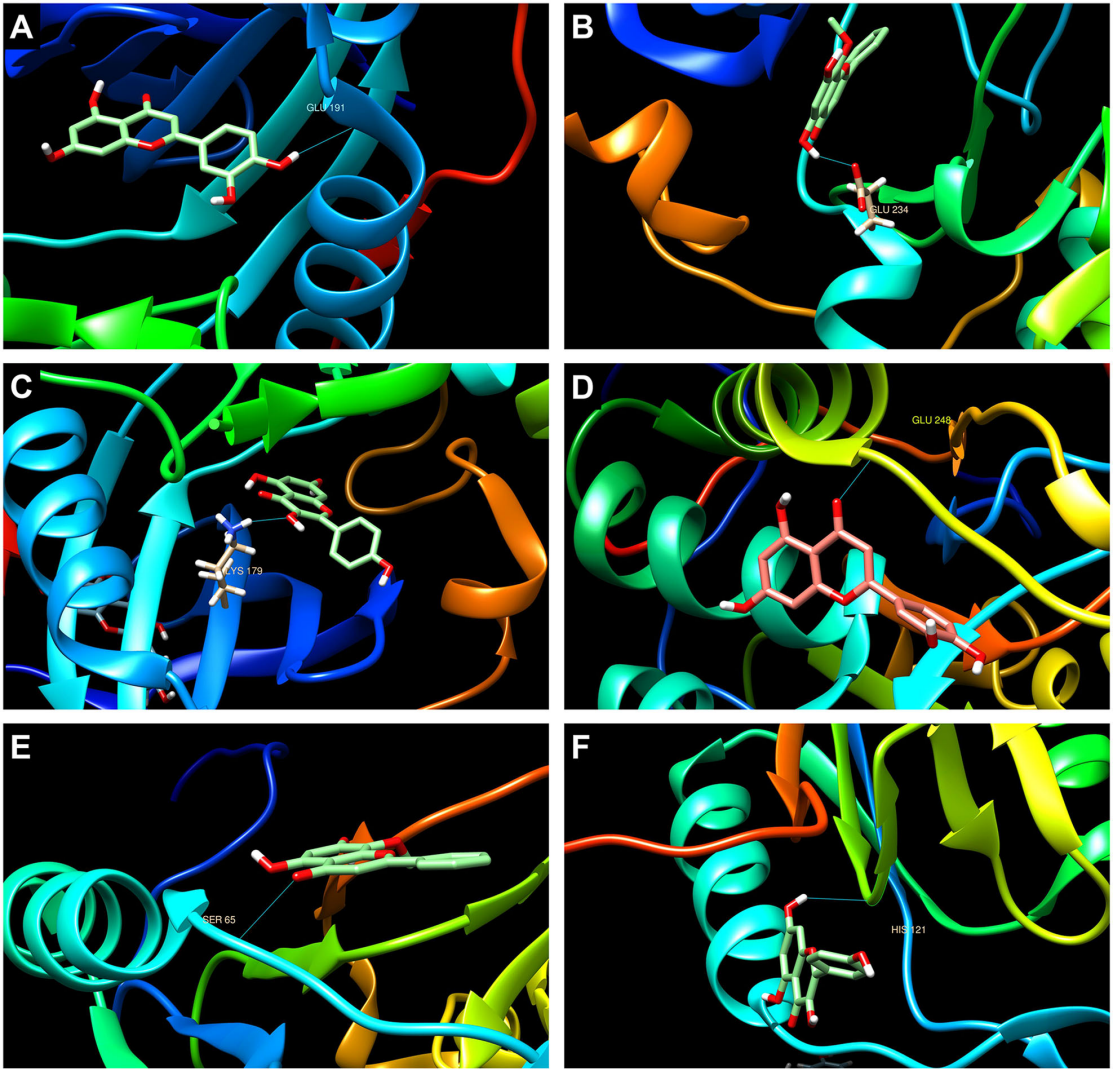
Compound-major target molecular docking. (A–C) The interaction mode of AKT1 with luteolin, wogonin, and kaempferol, respectively. (D–F) represented the interaction mode of CASP3 with luteolin, wogonin, and kaempferol, respectively.

Firstly, AKT1 interacted with luteolin, wogonin, and kaempferol. The docking scores of luteolin, wogonin, and kaempferol with AKT1 were 8.0, 7.7, and 4.5, respectively (Figure 7(A–C)). Meanwhile, CASP3 also interacted with luteolin, wogonin, and kaempferol. The docking scores of luteolin, wogonin, and kaempferol with CASP3 were 7.5, 4.8, and 4.5, respectively (Figure 7(D–F)).

### LUT treatment increased the viability and inhibited the apoptosis of ADR-treated MPC-5 cells *via* regulating AKT1 and CASP3

**Figure 8.**
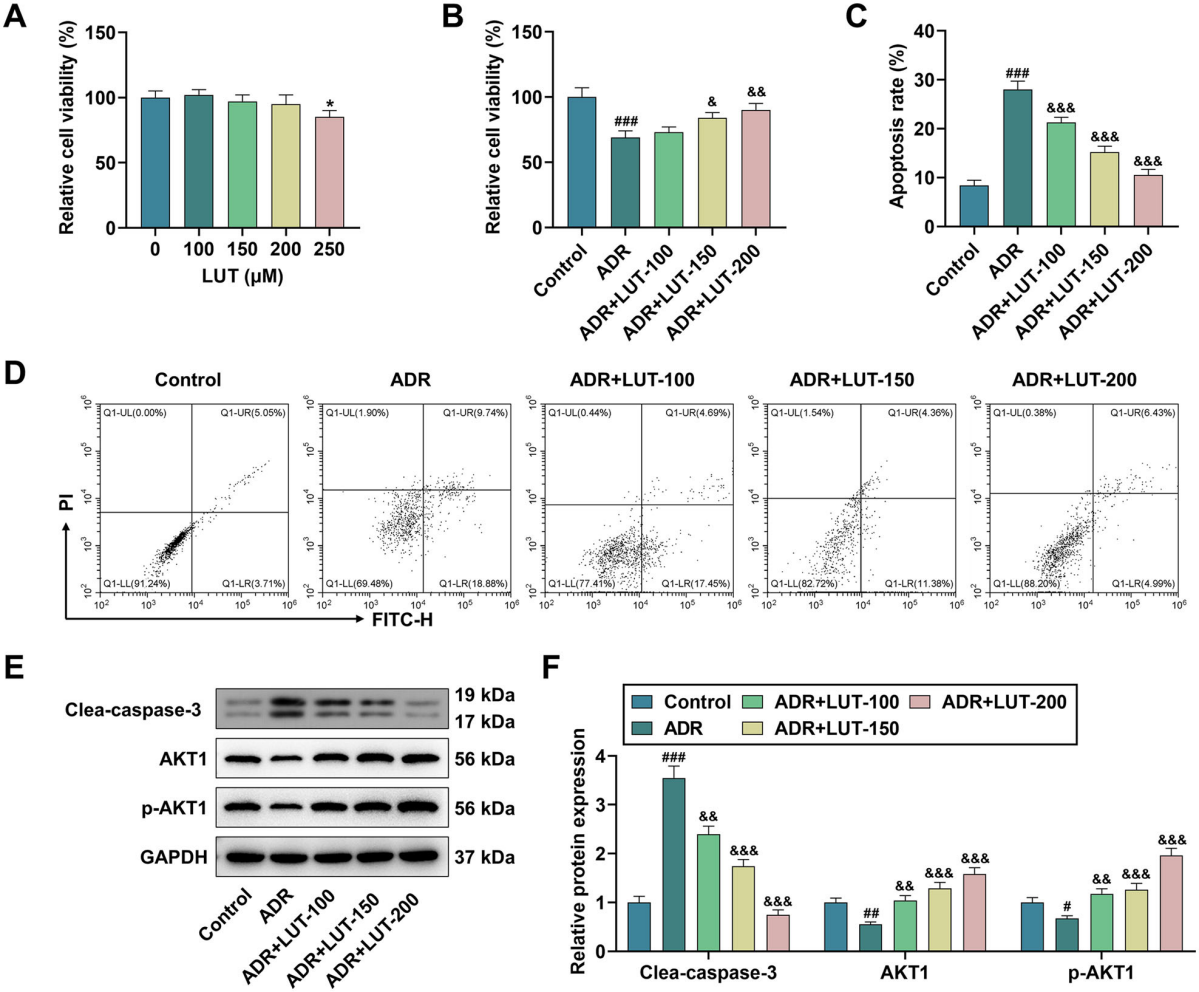
Effects of luteolin (LUT) treatment on the viability and apoptosis of ADR-treated MPC-5 cells as well as the protein expressions of AKT1 and CASP3. (A) The viability of MPC-5 cells in the LUT (0, 100, 150, 200, 250 μM) groups was determined by cell counting kit-8 (CCK-8) assay. (B) CCK-8 assay was also performed again to detect the cell viability in the control, adriamycin (ADR), ADR + LUT-100, ADR + LUT-150, and ADR + LUT-200 groups. (C–D) The apoptosis rate of MPC-5 cells in the control, ADR, ADR + LUT-100, ADR + LUT-150, and ADR + LUT-200 groups was measured by flow cytometry. (E–F) The protein expressions of cleaved (Clea)-caspase-3, AKT1, and phosphorylated (p)-AKT1 were determined by Western blot, with GAPDH serving as the loading control.

According to the results of CCK-8 assay, 100, 150, and 200 μM of LUT had no significant effect on the viability of MPC-5 cells, while 250 μM of LUT led to the obvious decrease in cell viability (*p* < 0.05, Figure 8(A)). Next, we found that ADR treatment impaired the viability yet promoted the apoptosis of MPC-5 cells (*p* < 0.001, Figure 8(B–D)), and these trends were reversed by LUT in a dose-dependent manner (*p* < 0.05, Figure 8(B–D)). In addition, the protein expression of Clea-caspase-3 was increased but that of AKT1 or p-AKT1 was decreased following the treatment of ADR (*p* < 0.05, Figure 8(E–F)). Compared with those in the ADR group, Clea-caspase-3 protein expression level was lowered but the levels of AKT1 and p-AKT1 were elevated in the ADR + LUT (100, 150, 200) groups (*p* < 0.01, Figure 8(E–F)).

## Discussion

In this study, there were 181 active components and 186 targets of GYD screened by TCMSP. Additionally, we screened 518 potential targets of FRNS by GeneCards. Following the intersection of these targets, 51 latent targets were discovered to be associated with both active ingredients of GYD and FRNS. Then, we established a D-C-D-T network and PPI network. Among them, 10 targets, such as AKT1, CASP3, IL6, etc, had higher degree values and were identified as potential core targets for GYD in the treatment of FRNS. Based on the enrichment analysis, we found that GYD could play a potential role in the treatment of FRNS by intervening multiple signaling pathways.

For D-C-D-T network analysis, the top 3 effective chemical components ranked by degree value were luteolin, wogonin, and kaempferol. Luteolin exists in a large number of plants and has a good therapeutic effect on acute and chronic nephropathy [24,25]. The report of Meng XM et al. exhibited that wogonin alleviates cisplatin-triggered acute kidney injury *via* repressing RIPK1-mediated necroptosis [26]. Some scholars also reported that luteolin and wogonin are the top-ranking components in Yiqi Huoxue Decoction, which have the potential to alleviate podocyte injury, enhance renal function and improve adriamycin-induced nephrotic syndrome [27]. Kaempferol, also known as thymosin III, belongs to flavonoids and widely exists in various fruits, vegetables, and beverages, manifesting a pivotal function in treating and alleviating the symptoms of kidney diseases. For example, kaempferol effectively impedes calcium oxalate crystal-mediated kidney damage and crystal deposition through the modulation of AR/NOX2 signaling [28]. In addition, the immunoregulation activity of kaempferol has been revealed already, which can polarize macrophages to M2 phenotype [29]. Therefore, luteolin, wogonin, and kaempferol may exhibit key roles in the treatment of FRNS using GYD.

The STRING database was applied to perform PPI network analysis on the 51 selected intersected targets, among which the top 3 targets of degree were AKT1, CASP3, and IL6. AKT1, a member of the serine/tyrosine family, is a critical downstream molecule in the PI3K/AKT/mTOR pathway, and is involved in significant immune pathways such as cytokine activation [30]. An existing report unveiled that the deletion of AKT1 accelerated kidney fibrosis through the activation of TGF-β1/STAT3 signaling in a mouse model of unilateral ureteral obstruction (UUO) [31]. Xiaomin Wen et al. described the involvement of PI3K/AKT signaling pathway in the therapeutic effect of *Astragali Radix* on nephrotic syndrome [13]. Meanwhile, CASP3 expression in renal issues was up-regulated based on the observation in the adriamycin-triggered nephrotic syndrome rats [32]. Also, IL6 expression in renal issues was extremely intensified in UUO-triggered chronic kidney disease [33]. Besides, another study has suggested the pathogenic role of IL6 in children with INS [34]. Thus, AKT1, CASP3, IL6, etc. can be used as latent therapeutic targets for FRNS.

In the next research, we performed GO and KEGG enrichment analyses. The intersection targets were mainly enriched in processes like muscle cell proliferation, membrane raft, cytokine receptor binding, and signaling pathways such as the AGE-RAGE signaling pathway in diabetic complications, lipid, and atherosclerosis, TNF signaling pathway, etc. AGE-RAGE signaling pathway can trigger a series of inflammation, leading to islet cell damage and insulin resistance, which is tightly correlated with the occurrence of kidney-related diseases [35]. The main effector molecule in the TNF pathway is TNF-α, which binds to tumor necrosis factor receptor (TNFR) to produce JNK and NF-κB, thereby inducing inflammatory response and insulin resistance [36,37]. Moreover, the combination of TNF-α and IFN-γ in the induction of podocytes can mimic steroid resistant nephrotic syndrome *in vitro [*38]. Collectively, the above processes and signaling pathways can serve as the potential mechanisms of GYD in the treatment of FRNS.

Then, the first two targets (AKT1 and CASP3) with high scores were singled out from 51 latent targets, followed by molecular docking with 3 active components of GYD (luteolin, wogonin, and kaempferol). It turned out that AKT1 or CASP3 interacted with luteolin, wogonin, and kaempferol, respectively, unveiling that AKT1 and CASP3 may be the key targets of luteolin, wogonin, and kaempferol against FRNS. Furthermore, cell experiments showed that luteolin treatment was able to enhance the viability and inhibit the apoptosis of FRNS-modeled cells *via* regulating AKT1 and CASP3, which further validated our previous results from the bioinformatics analysis.

## Conclusions

In conclusion, the chemical components like luteolin, wogonin, and kaempferol in GYD, the core targets including AKT1, CASP3, and IL6, the processes such as muscle cell proliferation, membrane raft and cytokine receptor binding, as well as the signaling pathways such as AGE-RAGE signaling pathway in diabetic complications, lipid, and atherosclerosis, TNF signaling pathway, etc., are the potential mechanisms accounting for the effects of GYD in the treatment of FRNS. The innovation of this paper is our first discovery of active compounds, latent targets, and molecular mechanisms of GYD in FRNS, which provides us with a better understanding on the therapeutic role of GYD on FRNS. Despite these considerable findings, there are still some limitations in this study that should be addressed. Firstly, we may ignore the roles of some compounds and target genes in FRNS due to the continuous updates of the public databases. Secondly, additional animal experiments and clinical experiments are required to further support and verify the specific mechanism of GYD in the treatment of FRNS.

## Data Availability

The analyzed data sets generated during the study are available from the corresponding author on reasonable request.
